# PhenomiR: a knowledgebase for microRNA expression in diseases and biological processes

**DOI:** 10.1186/gb-2010-11-1-r6

**Published:** 2010-01-20

**Authors:** Andreas Ruepp, Andreas Kowarsch, Daniel Schmidl, Felix Buggenthin, Barbara Brauner, Irmtraud Dunger, Gisela Fobo, Goar Frishman, Corinna Montrone, Fabian J Theis

**Affiliations:** 1 Institute for Bioinformatics and Systems Biology (MIPS), Helmholtz Center Munich - German Research Center for Environmental Health (GmbH), Ingolstädter Landstraße 1, D-85764 Neuherberg, Germany; 2 Department of Mathematics, University of Technology Munich, Boltzmannstraße 3, D-85747 Garching, Germany

## Abstract

In recent years, microRNAs have been shown to play important roles in physiological as well as malignant processes. The PhenomiR database http://mips.helmholtz-muenchen.de/phenomir provides data from 542 studies that investigate deregulation of microRNA expression in diseases and biological processes as a systematic, manually curated resource. Using the PhenomiR dataset, we could demonstrate that, depending on disease type, independent information from cell culture studies contrasts with conclusions drawn from patient studies.

## Abstract 2

PhenomiR is a comprehensive database of 542 studies reporting deregulation of miRNAs allowing large-scale statistical analysis of miRNA expression changes.

## Rationale

MicroRNAs (miRNAs) are approximately 22-nucleotide endogenous RNAs predicted to regulate the expression of most mammalian genes [1]. Since the discovery of miRNAs in *Caenorhabditis elegans*, the influence of these regulatory RNAs on cellular processes has been established in a large variety of metazoa [2]. Accordingly, individual studies as well as large-scale endeavors have detected a growing number of miRNAs [3,4], up to 695 in human according to miRBase release 12.0 [5]. A proteome study that investigated the influence of the abundance of a single miRNA on cells found that the mode of regulation occurs through modulation of protein expression rather than as a binary off-switch [6].

However, the potential of deregulated miRNA expression to cause severe impairments has already been demonstrated in the early days of microRNA research [7]. In 2004, it was shown that deregulated miRNA expression is associated with human diseases such as lung cancer [8]. One year later, Lu *et al*. [9] analyzed miRNA expression in cancer types and observed that miRNA profiling is a more reliable indicator for cancer than mRNA expression profiles. In the meantime, additional studies have demonstrated that miRNAs are significant indicators for specific diseases and can, for example, be used to create decision trees differentiating cancer types solely by miRNA expression profiles [10,11]. In recent years, deregulated expression of miRNA has also been found to be associated with human diseases such as cardiomyopathy, muscular disorders and neurodegenerative diseases [12-14]. The samples used for these studies stem from biopsies of patients or cell cultures, which are used as easily tractable experimental models. Besides diseases, microRNAs are also known to have functional roles in eukaryotic organisms. MicroRNA-mediated gene silencing was shown to be involved in a number of cellular processes, such as cell growth, larval development and B-cell differentiation [15,16,7].

Due to the increasing amount of data in miRNA research, several resources have been established, covering topics such as experimentally validated miRNA targets (Tarbase [17]), and prediction of miRNA targets (Targetscan [18], PITA [19], PicTar [20]) or serving as miRNA repositories (miRBase [5]).

**Figure 1:**
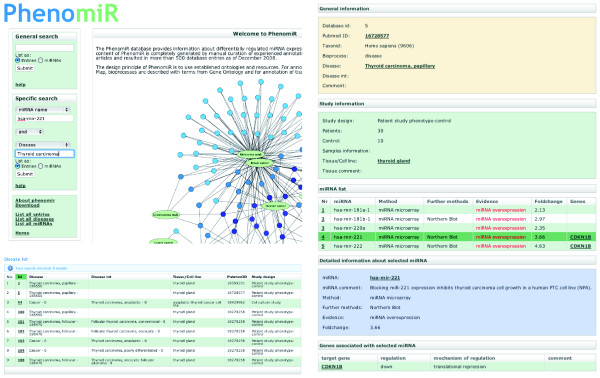
**Overview of the PhenomiR web page, the search options, search results and a database entry**.

In order to provide a comprehensive overview of differentially regulated miRNA expression data in diseases and general biological processes, we generated the PhenomiR database. We aim at high data quality by manual annotation by experienced biocurators. PhenomiR provides an in-depth annotation of the studies, not only including information like the mode of miRNA expression (up or down) and the miRNA detection method, but also data such as the quantitative fold-change of miRNA expression, the sample size and the origin of the samples (patients or cell culture) analyzed (Figure 1), which are not available from any existing resource. This comprehensive repository allows for the first time a large-scale statistical analysis of aspects such as genomic localization of deregulated miRNAs or the influence of sample origin. Using PhenomiR data from cell culture studies and patient studies, we found that, depending on the disease type, independent information from cell culture studies is in conflict with conclusions drawn from patient studies. Furthermore, a systematic analysis of 94 diseases shows for the first time that deregulated microRNA clusters are significantly overrepresented in the majority of investigated diseases (approximately 90%) compared to singular microRNA gene products.

## Results and discussion

### Database contents

In recent years, a wealth of studies published in the scientific literature has investigated deregulation of miRNA expression in diseases and other biological processes. PhenomiR provides a repository that offers all the scattered information about miRNA expression in a structured and uniform format. This allows users to perform individual queries for specific miRNAs and diseases as well as to use the complete dataset for large-scale statistical analyses. All information in PhenomiR is extracted from published experiments and has been manually curated. The literature reference for each database entry is annotated as a PubMed identifier and is hyper-linked to PubMed in the web frontend. Each individual entry of the database refers to an instance of a publication describing a specific disease or bioprocess (Figure 1). Currently, PhenomiR documents data from 296 articles that describe 542 studies. This dataset includes 11,029 data points, each representing one deregulated miRNA in an experiment.

**Figure 2:**
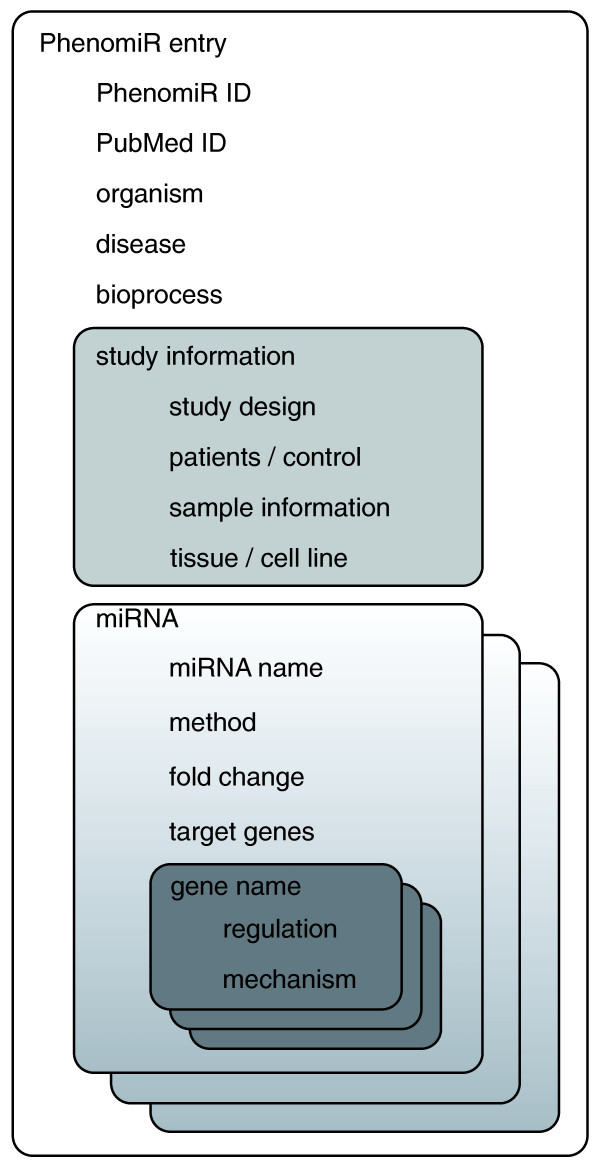
**Overview of a PhenomiR entry structure**.

A design principle of PhenomiR is to use well-established ontologies and resources. As miRBase is the primary resource for miRNA annotation and nomenclature, we use the miRBase identifiers and nomenclature for annotation of miRNAs. In order to enable convenient analysis of the dataset, miRNA designations from previous nomenclature releases were mapped to miRBase release 12.0 [5]. For annotation of diseases we use information from the Online Mendelian Inheritance in Man (OMIM) Morbid Map [21]. The OMIM Morbid Map is an alphabetical list of diseases described in OMIM, including their corresponding cytogenetic locations. In contrast to disease vocabularies like Disease Ontology (DO) or MeSH (Medical Subject Heading) disease categories, the widely popular OMIM classification scheme contains additional information about the disease, such as clinical features, population genetics and genes that are experimentally shown to be involved in the respective disease. If no appropriate OMIM disease term is available for the annotation of a disease (currently the case for 20.7% of studies), we introduce additional terms like 'dermatomyositis' and 'thyroid carcinoma, medullary'. In addition to the OMIM terms, PhenomiR annotates Morbid Map-associated higher-level disease classes, such as cancer or cardiovascular, which were introduced by Goh *et al*. [22]. In this system, each annotated disease from the Morbid Map is associated with one of 22 disease classes. miRNA expression analyses of biological processes are predominantly performed for developmental processes and responses to conditions like folate starvation. For the annotation of biological processes we assign terms from Gene Ontology [23]. Cell lines or tissues (Figure 2) that were used as samples in the analyses are annotated using the BrendaTissueOntology (BTO) [24].

**Figure 3:**
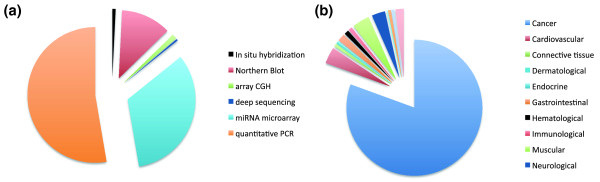
**Fraction of annotated miRNA detection methods and diseases in PhenomiR**. **(a) **Distribution of detection methods for all disease entries in PhenomiR. **(b) **Distribution of disease entries in PhenomiR.

In addition to the sample information, PhenomiR provides the experimental methods used for miRNA expression analyses: to a large extent, expression studies of miRNAs have been performed with microarrays (29% of all miRNA phenotype correlations). Other methods, such as RT-PCR (47%) and Northern blot (10%), are also used to reconfirm the results for selected miRNAs (Figure 3a). Information about differential expression of miRNAs in PhenomiR is given as the qualitative attributes 'miRNA overexpression' or 'miRNA downregulation'. In most articles (75%) authors also publish quantitative results. This information allows discrimination between marginally and significantly deregulated miRNAs. If such information is available, quantitative data (as fold-change) are additionally annotated in PhenomiR. Data content from miRNA expression studies curated in PhenomiR show a high heterogenity in the amplitude of fold-change and the available measurements. Studies like that of Nikiforova *et al*. [25] present only few values considered to be significant by the authors, whereas in an analysis of melanoma and neural system tumor syndrome 222 values are presented [26]. Accordingly, the extent of maximum miRNA deregulation lies in a range from 1.42-fold in a renal cell carcinoma study [27] to 5,997-fold for acute lymphoblastic leukemia [28]. Therefore, we do not set arbitrary thresholds for the numbers of deregulated miRNAs or the fold-changes but present the data as they are provided by the scientists, leaving possible filtering and thresholding or weighting to any later analysis.

In case studies containing analyses of putative target genes from significantly deregulated miRNAs, verified target genes are annotated. The annotation includes gene name and identifier of the target gene, the effect on gene product expression (up- or down-regulation) as well as the mechanism of regulation, for example, transcriptional repression or translational inhibition (Figure 1).

A survey about the PhenomiR dataset reveals that cancers are by far the most thoroughly investigated diseases (81%) followed by muscular (4.3%) and cardiovascular (4.1%) disorders (Figure 3b). The largest number of cancer studies is devoted to leukaemia (16.7%), colorectal cancer (10.6%) and breast cancer (9.5%).

PhenomiR is the largest publicly available resource of miRNA deregulation in diseases and biological processes, providing 11,029 data points (miR2Disease: 2,663 data points) and 572 miRNAs (miR2Disease: 347 miRNAs) as of September 2009. Out of 542 PhenomiR entries, 90 provide information about miRNA expression in biological processes such as cardiac muscle development or eye development, which are not available from any other existing database. In comparison to resources like miR2Disease [29] and HMDD [30], PhenomiR provides comprehensive experiment information such as fold-change of miRNA dysregulation, cohort information and study design. Moreover, we particularly focused on the thorough use of ontologies, which are invaluable for in-depth statistical analysis and further exploitation of the data as shown in the analyses below. Especially in publications presenting all data on deregulated miRNAs, fold-change information allows a threshold to be set in order to separate marginally from significantly deregulated miRNAs. Cohort information specifies the number of patients analyzed in a study and thus determines the statistical significance of the data. Data from cell culture studies and patient studies are identified by the study design information. Without this information the first data analysis shown below would not have been possible. Finally, to our knowledge, manually annotated data about differential miRNA regulation in bioprocesses are not found in any other publicly available database. PhenomiR is freely accessible and the data can be downloaded as tab-delimited text files (see also Additional file 1). New content releases for PhenomiR will appear every half year.

### Search options and predefined datasets

In order to obtain an overview of the PhenomiR dataset, the web page links to three lists that display: all entries; all diseases; and all annotated miRNAs (Figure 1). In addition, statistical information about the number of database entries, most frequently annotated miRNAs, and so on are provided on the front page. Currently, 567 different miRNAs were found to be deregulated in at least one entry.

For queries, PhenomiR offers two search options, a 'General search' as well as a 'Specific search' (Figure 1). The 'General search' performs simultaneous queries across several attributes like 'miRNA name', 'disease' or 'gene name'. This is optimized for searches where comprehensiveness rather than specificity is required. The results can be displayed either as respective entries or associated miRNAs. The 'Specific search' allows the selection of individual annotated attributes shown in a pull-down menu. Additionally, specific searches can be combined by using the logic operators AND, OR and NOT. As in the 'General search', results can be displayed as a list of database entries. Another way to depict the results is to generate a list of all miRNAs found in any of the corresponding studies. Results of both search options are linked to the respective entries.

To demonstrate the additional value of the comprehensive annotation in PhenomiR, we investigated the influence of differentially regulated genomic microRNAs on diseases from a large-scale statistical point of view.

### Differences between disease-associated microRNA expression in patients and cell lines

Cell lines have been established in life sciences as easy to manipulate model systems for the study of cellular processes. However, studies using both *in vitr*o and *in vivo *systems have shown that the results from each - for example, in cancer - do not always correlate. In previous studies differences in gene expression patterns between cell lines and their fresh-frozen tissue counterparts have been observed [31]. Accordingly, analysis of DNA copy number alterations between cell lines and fresh tissue revealed recurring deviations in cell lines [32]. Cell cultures are also frequently used to investigate differential miRNA expression in cellular systems. In PhenomiR, we have collected 119 *in vitro *studies of miRNA expression in various diseases revealing implications for the prognosis of diseases. With respect to the discrepancies between cell cultures and living organisms mentioned above, we asked whether cell cultures are reliable disease models for the analysis of differential miRNA expression.

**Figure 4:**
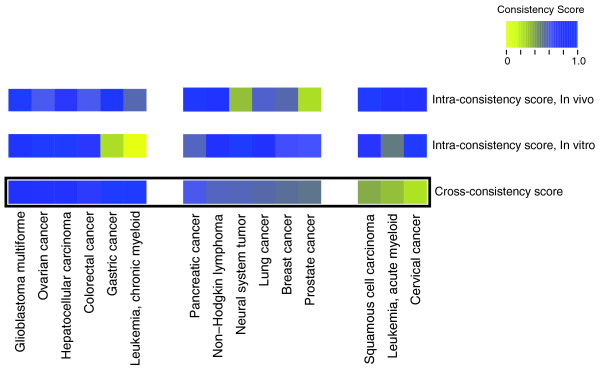
**Comparison of consistencies in expression profiles between *in vivo *and *in vitro *experiments**.

In order to analyze the concordance of *in vivo *and *in vitro *data, we extracted disease information from PhenomiR with sufficient miRNA annotation for both study designs. We first compared the consistency of miRNA annotation within each disease for both *in vivo *and *in vitro *experiments. This was done by means of an intra-consistency score, defined as the fraction of miRNAs showing a concordant expression pattern within a disease annotated by at least two experiments. In a second step, we computed the cross-consistency score between *in vivo *and *in vitro *data as the fraction of miRNAs showing the same expression pattern between these two study designs. Figure 4 shows the obtained consistency scores for 15 diseases that had sufficient data coverage in PhenomiR. Only 6 out of 15 diseases (glioblastoma, ovarian cancer, hepatocellular carcinoma, colorectal cancer, gastric cancer and chronic myeloid leukemia) show the expected high cross-consistency (73 to 100%) between *in vivo *and *in vitro *experiments. On the other hand, we found six diseases (pancreatic cancer, non-Hodgkin lymphoma, neural system tumor, lung cancer, breast cancer and prostate cancer) with only moderate cross-consistency scores (51 to 61%). Analyzing the corresponding *in vivo *and *in vitro *data, we obtained high intra-consistency scores, which indicate a high homogeneity within these experiments. However, the resulting cross-consistency scores are rather low, implying limited relevance of *in vitro *experiments for those diseases. Finally, we also found three diseases (squamous cell carcinoma, acute myeloid leukemia (AML) and cervical cancer) with low cross-consistency (24 to 38%), revealing severe discrepancies between cell culture experiments and patient studies. High intra-consistency scores corroborate the significance of this observation and exclude the possibility that the results stem from different experimental conditions.

These findings could possibly arise as an artifact of the selection of cell cultures or subtypes of diseases investigated in the published studies. Indeed, this is not the case, as can be seen from the example of AML. AML is, according to the French-American-British (FAB) classification system, divided into eight subtypes, M0 through to M7, based on the type of cell from which the leukemia developed and its degree of maturity. All AML cell culture studies have been performed with NB-4 cells and HL-60 cells, which are both from the M3 subtype (promyelocytic or acute promyelocytic leukemia). In contrast, patient studies from different AML subtypes are annotated in PhenomiR. For our analysis, data from the respective patient studies has been pooled in order to obtain a statistically sufficient amount of data. However, comparison of cell culture studies with data from Saumet *et al*. [33], which also analyzed patients with AML from the M3 subtype, confirm that our findings hold true for similar AML subtypes. In cervical cancer the two patient studies did not classify specimen according to the World Health Organization classification or the Bethesda System. However, the three different cell lines that were used for *in vitro *studies exhibited consistent results (100%). In conclusion, these two examples show that our findings are not distorted by the origin of the samples that were used in the studies.

Recent studies have shown that miRNA expression profiles have a high prognostic potential in disease classification and that it is even possible to build decision trees in order to differentiate cancer tissue origins [10,11]. However, our large-scale analysis including data from more than 413 surveys has shown that data from *in vitro *and *in vivo *studies correlate for diseases like pancreatic cancer and ovarian cancer, but display significant inconsistencies in squamous cell carcinoma and cervical cancer. Discrepancies between experimental results from organisms and cell cultures could occur for two reasons. Most notably, the cell line immortalization process has been implicated as a source of cytogenetic changes [34]. In addition, multiple growth passages, to which commercially available cell lines are routinely subjected, have been shown to be associated with random genomic instability [35]. These observations and the results of our study show that the potential of cell cultures in the investigation of miRNA expression in diseases is limited. As a consequence, the suitability of cell cultures has to be verified for each disease and cell line before using such data as a tool for the prognosis of diseases in human.

### microRNA clusters are significantly overrepresented in most investigated diseases

While creating the PhenomiR database we found individual studies that investigated the impact of not only deregulated single miRNAs but also miRNA clusters, such as miR-17-92, miR-106b-93-25 and miR-222-221, on diseases, especially cancer [36,37]. Using the comprehensive dataset from our PhenomiR database we asked whether the impact of miRNA clusters on diseases is restricted to only a few examples or whether miRNA clusters significantly correlate with the pathobiology of diseases. According to release 12.0 of mirBase, 695 miRNA genes have been detected in the human genome so far [5]. Analysis of the genomic distribution of miRNAs shows that it is strongly biased towards neighborhoods on chromosomes. Given a maximum distance of 5 kb, about 34% of human miRNAs appear as miRNA clusters of at least two members, leading to 62 miRNA clusters. Microarray profiling of miRNAs has shown that neighboring miRNAs within a distance of up to 50 kb are frequently co-expressed [38]. It can be assumed that miRNA clusters are not only often jointly expressed but also act in a concerted way on interrelated cellular functions [36].

First, we systematically analyzed the homogeneity of expression patterns within miRNA clusters. In order to determine the concordance of expression, we excluded those clusters from further analysis having less than half of all miRNAs annotated in PhenomiR, leading to 47 remaining clusters. The clusters are denoted as exhibiting a homogeneous expression pattern if all annotated miRNAs are either up- or downregulated. In total, disease-associated clusters revealed homogeneous expression patterns for 77% of all annotated diseases, which confirms the hypothesis of co-expression of miRNA clusters. For example, cluster mir-221-222 shows a consistent expression pattern in 93% of the diseases (Additional file 2).

As some of the investigated diseases show an extremely unidirectional expression pattern - that is, almost all annotated miRNAs are either upregulated or downregulated - we might find homogeneous patterns even by chance. In order to take this effect into account, we created a null model by randomly linking miRNA expression patterns (10,000 times within each disease). In total, 23 clusters (50%, *P*-value < 0.05) showed a significantly higher homogeneity pattern in all annotated diseases compared to that expected by chance (Additional file 2). These clusters exhibit a homogeneous expression pattern in at least 87% of all annotated diseases.

**Figure 5:**
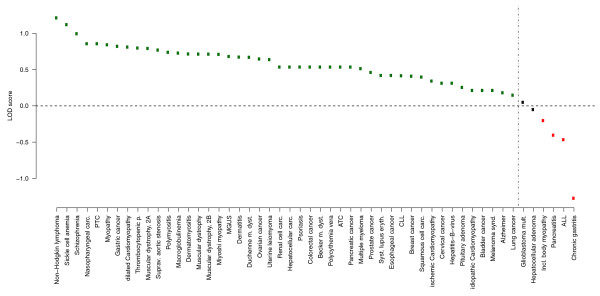
**miRNA cluster enrichment in human diseases**. For each disease the log-odds (LOD) score is plotted. There is an enrichment of miRNA cluster members for 46 diseases (88.5%). miRNA cluster members are, on average, 1.4 times (LOD = 0.58) enriched compared to random. Green points depict enriched diseases (at least LOD = 0.15 for lung cancer). Black points indicate diseases without enrichment compared to random and red points depict disorders with few deregulated cluster members. For abbreviations of disease names see Additional file 13.

To investigate the association of miRNA clusters with human diseases, we estimated the enrichment of miRNA clusters in disease-associated miRNAs. Analysis from articles restricted to only a few miRNAs could introduce an overestimation of disease association with miRNA clusters. In order to avoid this bias, we chose only data from patient studies using miRNA microarrays, since microarrays are standardized tools that aim to cover the most comprehensive dataset of known miRNAs. For the estimation of cluster enrichment we used a log-odds score (LOD): we calculated the fraction of disease-associated miRNAs within a cluster for each disease and divided this number by the background frequency of 34% (Figure 5). We found enrichment for 46 out of 52 (88.5%) diseases (*P*-value = 6.1 × 10^-3^). Within these 46 diseases, miRNAs located in clusters are, on average, 1.4 times (LOD = 0.58) enriched compared to random. However, it may be argued that polycistronic miRNA loci are more likely associated with diseases because multiple combinations of miRNAs could possibly generate a phenotype, that is, only one miRNA of a cluster may act as the causative while the others act as 'bystanders'. In order to address this question, we considered miRNA clusters as single loci and calculated the enrichment of polycistronic miRNA loci for each disease (see Materials and methods). We found that polycistronic loci are, on average, 3.5 times more disease-associated than expected by chance (Additional file 3), whereas differentially expressed single miRNA loci are not enriched in diseases (Additional file 4). In conclusion, both analyses show a significant enrichment of clustered miRNAs in diseases regardless of whether the single miRNA members are used for the analysis or the cluster is viewed as one locus. Thus, it is highly unlikely that only one miRNA of a cluster is associated with a disease. Indeed, experimental analyses show that different miRNAs from miRNA clusters act synergistically [36,39].

These results show that deregulation of miRNA clusters in diseases is obtained not just in a few examples but appears to occur systematically in the vast majority of human diseases investigated in our analysis. Although studies of miRNA expression in diseases are dominated by various types of cancer (Figure 3), comparable results are found if cancer and non-cancer diseases are examined separately (Additional file 5). The lower deregulation of single miRNAs is probably due to the fact that miRNAs do not act as binary off-switches but rather modulate protein expression [6]. For instance, the response to altered mir-223 expression on the human proteome indicates that, for most interactions, miRNAs act as rheostats to make fine-scale adjustments to protein output [6]. As shown above, deregulation of miRNA clusters affects expression of several miRNAs at the same time. A concerted action of several miRNAs on a common target or pathway has a much higher potential to influence cellular processes. In fact, for several specific clusters such a synergistic regulatory effect has been shown. Overexpression of miR-200 miRNA clusters in NMuMG cells hindered epithelial-mesenchymal transition by enhancing E-cadherin expression through direct targeting of *ZEB1 *and *ZEB2*, which encode transcriptional repressors of E-cadherin [39]. In gastric cancer, two miRNA clusters, miR-106b-93-25 and miR-222-221, were found to suppress different cell-cycle inhibitors [37]. Such results are further corroborated by *in silico *analyses of target genes of members of miRNA clusters [40]. These experimental findings and the systematic correlation of miRNA cluster deregulation with human disease shown here strongly support the idea that a coordinated regulatory effect is a general attribute of miRNA clusters. The pivotal role of miRNA clusters in miRNA-based gene silencing found in human diseases suggests that effective treatment of various diseases may require a combinatorial approach to target not singular miRNAs but rather miRNA clusters.

## Materials and methods

### Comparison between *in vivo *and *in vitro *experiments

To evaluate the expression consistency of miRNAs *in vivo*, we first allocated all *in vivo *expression profiles in PhenomiR to the corresponding diseases. Within each disease, these entries were grouped by miRNAs and all groups containing less than two entries were discarded. Subsequently, we checked for consistent expression profiles - that is, all entries for a specific miRNA must show the same expression (either down- or up-regulation) to be counted as consistent. The intra-consistency score for *in vivo *or *in vitro *experiments is defined for every disorder to be the fraction of miRNAs with consistent expression patterns throughout all allocated entries of a group and all miRNAs involved in the disease. For the estimation of the cross-consistency score we grouped all miRNAs with consistent expression profiles in both study designs (*in vivo *and *in vitro*) for that specific disease. Additionally, we added those miRNAs to the groups that contained only one entry in the *in vivo *and *in vitro *experiments, respectively. The cross-consistency score for comparison of *in vivo *and *in vitro *experiments was then calculated as the fraction of miRNAs showing a consistent expression pattern compared to the total number of miRNAs for each disease.

### Human miRNA cluster data

A miRNA cluster is defined as set of miRNAs in which each member has at least one other member of the same set within 5 kb according to chromosomal locations. Chromosomal positions for all human miRNAs were obtained from mirBase (release 12.0). In total, we obtained 62 human miRNA clusters containing, in sum, 240 of 695 (34%) human miRNAs in miRBase. For results based on miRNA clusters defined by 10-kb and 50-kb thresholds, see Additional files 6, 7, 8, 9, 10, 11, 12 and 13).

### Analysis of homogeneous expression patterns within miRNA clusters

For the systematic analysis of coexpression of miRNA clusters, we considered all miRNAs associated with the particular diseases. miRNAs not belonging to any cluster and miRNAs of clusters of which at least half the members are not associated with the appropriate disease were discarded. For clusters containing only two members, both miRNAs had to be present. In total, we obtained 47 unique clusters. We defined a cluster to be homogeneous if all present members (which is at least half of all members) show the same expression pattern (either all up- or all downregulated). For each unique cluster we thereafter computed the homogeneous-fraction, that is, the fraction of co-expression throughout all obtained disease entries, and calculated a *P*-value for this fraction by the following sampling approach: for every disease entry the expression of all its associated miRNAs was distributed randomly within these miRNAs for 10,000 times, keeping the distribution of up- and downregulated miRNAs constant for each step. For each sampling step the homogeneous fraction over all disease entries was computed, which yields the *P*-value as the number of sampled homogeneous fractions exceeding the original homogeneous fractions divided by 10,000.

### Enrichment analysis of miRNA clusters in human diseases

For this part of the study only data from microarray experiments were taken into account in order to avoid a bias introduced by expression experiments investigating only a few miRNAs by, for example, RT-PCR. To measure the enrichment of cluster miRNAs compared to single miRNAs in human diseases, we set up a sampling algorithm based on log-odds (LOD) scores: for each disease, d, we calculated the number of cluster miRNAs, x_d_, and the number of non-cluster miRNAs, y_d_. The LOD score for disease d is then computed by:

where x_ovarall _denotes the number of the 240 human cluster miRNAs and y_overall _denotes the number of the 455 human miRNAs not contained in any cluster as obtained from mirBase (release 12.0). Note that x_overall _and y_overall _take into account all known human miRNAs, not just those annotated in PhenomiR. It can be easily seen that the LOD score for the enrichment of miRNAs not contained in any cluster computes to -LODd, where d is again the disease index. A positive LOD score indicates enrichment for cluster miRNAs compared to non-cluster miRNAs in a specific disease. For evaluation of the hypothesis of enrichment of cluster miRNAs throughout all human diseases we randomly shuffled the genomic position of all miRNAs in each disease 100,000 times and computed the fraction of cases where the number of sampled positive LOD scores was at least as high as the number of positive LOD scores obtained from the data. In addition, we considered miRNA clusters as single loci and calculated the enrichment of polycistronic miRNA loci by a LOD score: for each disease d we calculated the number of polycistronic miRNA loci, xd, and the number of single miRNAs, yd. The LOD score for disease d is then computed by:

where x_overall _denotes the number of the 62 human polycistronic miRNA loci and y_overall _denotes the number of the 455 human miRNAs not contained in any cluster as obtained from mirBase (Release 12.0).

### Statistical models in the Results and discussion section

All programs for this work were written in Python. Final data analysis and statistical models were done with the R statistical language.

## Abbreviations

AML: acute myeloid leukemia; LOD: log-odds score; OMIM: Online Mendelian Inheritance in Man.

## Authors' contributions

AR, AK, and FJT conceptualized the study, FB and FJT implemented the database and the web services, AK and DS analyzed the data, AR, BB, ID, GMF, GF, and CM performed the data curation of PhenomiR and AR, AK, DS, and FJT wrote the manuscript. All the authors have read and agreed to the manuscript.

## Supplementary Material

Additional file 1 The PhenomiR dataset. Click here for file

Additional file 2 T, number of diseases for which each cluster showing a homogeneous expression pattern; F, number of diseases for which each cluster shows no homogeneous expression pattern; Homogeneous-fraction, number of diseases for which each cluster shows a homogeneous expression pattern for each miRNA cluster as defined; *P*-value, estimate by randomly linking miRNA-expression patterns 10,000 times within each disease. Click here for file

Additional file 3 For each disease the log-odds (LOD) score is plotted. We found that polycistronic loci are on average 3.5 times (LOD = 1.83) more disease-associated than expected. For abbreviations of disease names see Additional file 13. Click here for file

Additional file 4 For each disease the log-odds (LOD) score is plotted. The LOD score for disease d is given by: LOD_d _= log_2 _((y_d_/(x_d _+ y_d_))/(y_overall_/(x_overall _+ y_overall_))) (see Materials and methods for a detailed description). We found that differentially expressed single miRNA loci are not enriched in diseases. Red points depict disorders with few deregulated single miRNA loci and black points indicate diseases without enrichment compared to random. For abbreviations of disease names see Additional file 13. Click here for file

Additional file 5 **(a) **miRNA cluster enrichment for cancer diseases. **(b) **miRNA cluster enrichment for non-cancer diseases. For each disease the log odds (LOD) score is plotted. Green points depict enriched diseases. Black points indicate diseases without enrichment compared to random and red points depict disorders with few deregulated cluster members. Click here for file

Additional file 6 T, number of diseases for which each cluster shows a homogeneous expression pattern; F, number of diseases for which each cluster shows no homogeneous expression pattern; Homogeneous-fraction, number of diseases for which each cluster shows a homogeneous expression pattern for each miRNA cluster as defined; *P*-value, estimate by randomly linking miRNA-expression patterns 10,000 times within each disease. Click here for file

Additional file 7 T, number of diseases for which each cluster shows a homogeneous expression pattern; F, number of diseases for which each cluster shows no homogeneous expression pattern; Homogeneous-fraction, number of diseases for which each cluster shows a homogeneous expression pattern for each miRNA cluster as defined; *P*-value, estimate by randomly linking miRNA-expression patterns 10,000 times within each disease. Click here for file

Additional file 8 For each disease the log odds (LOD) score is plotted. Order is based on LOD scores for polycistronic miRNA loci using a 5-kb distance threshold according to chromosomal locations. For abbreviations of disease names see Additional file 13. Click here for file

Additional file 9 The dashed line shows the background frequency, which is given by the number of the 62 human polycistronic miRNA loci, and the sum of polycistronic loci and the 455 human miRNAs that are not contained in any cluster as obtained from mirBase (release 12.0) using a 5-kb distance threshold according to chromosomal locations. For abbreviations of disease names see Additional file 13. Click here for file

Additional file 10 The dashed line shows the background frequency, which is given by the number of the 65 human polycistronic miRNA loci, and the sum of polycistronic loci and the 452 human miRNAs that are not contained in any cluster as obtained from miRBase (release 12.0) using a 10-kb distance threshold according to chromosomal locations. For abbreviations of disease names see Additional file 13. Click here for file

Additional file 11 The dashed line shows the background frequency, which is given by the number of the 72 human polycistronic miRNA loci, and the sum of polycistronic loci and the 445 human miRNAs that are not contained in any cluster as obtained from mirBase (release 12.0) using a 50-kb distance threshold according to chromosomal locations. For abbreviations of disease names see Additional file 13. Click here for file

Additional file 12 For each disease the log odds (LOD) score is plotted. Order is based on LOD scores for miRNA clusters using a 5-kb threshold. Click here for file

Additional file 13 Diseases used in this analysis and corresponding short names. Click here for file
