# Supplementary figures and images for: PhenomiR: a knowledgebase for microRNA expression in diseases and biological processes
Source: Genome Biol. 2010 Jan 20;11(1):R6. doi: 10.1186/gb-2010-11-1-r6 (PMC2847718; doi:10.1186/gb-2010-11-1-r6)

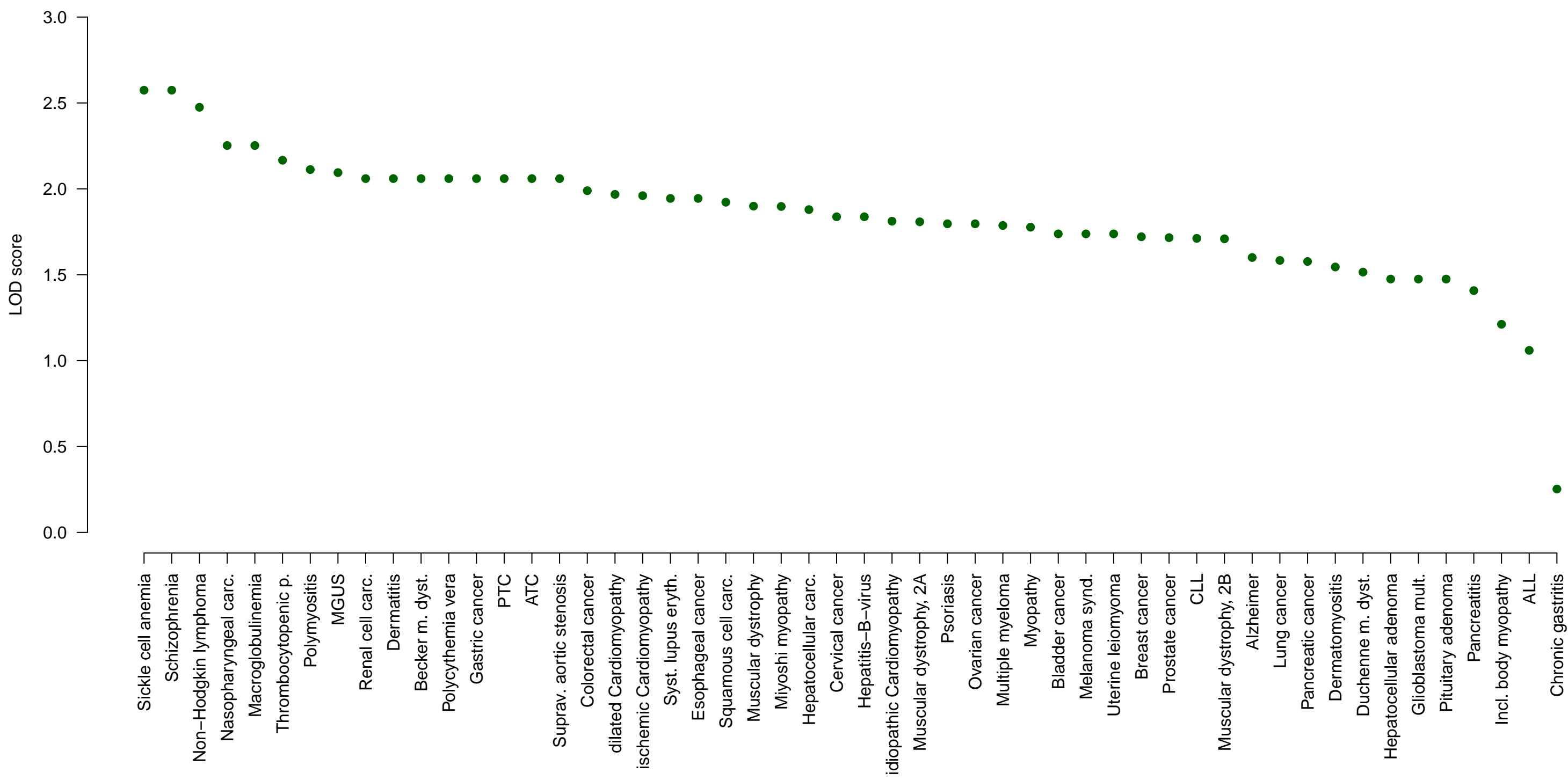

Supplement: Additional file 3 — For each disease the log-odds (LOD) score is plotted. We found that polycistronic loci are on average 3.5 times (LOD = 1.83) more disease-associated than expected. For abbreviations of disease names see Additional file 13. [file gb-2010-11-1-r6-S3.pdf]

LOD score

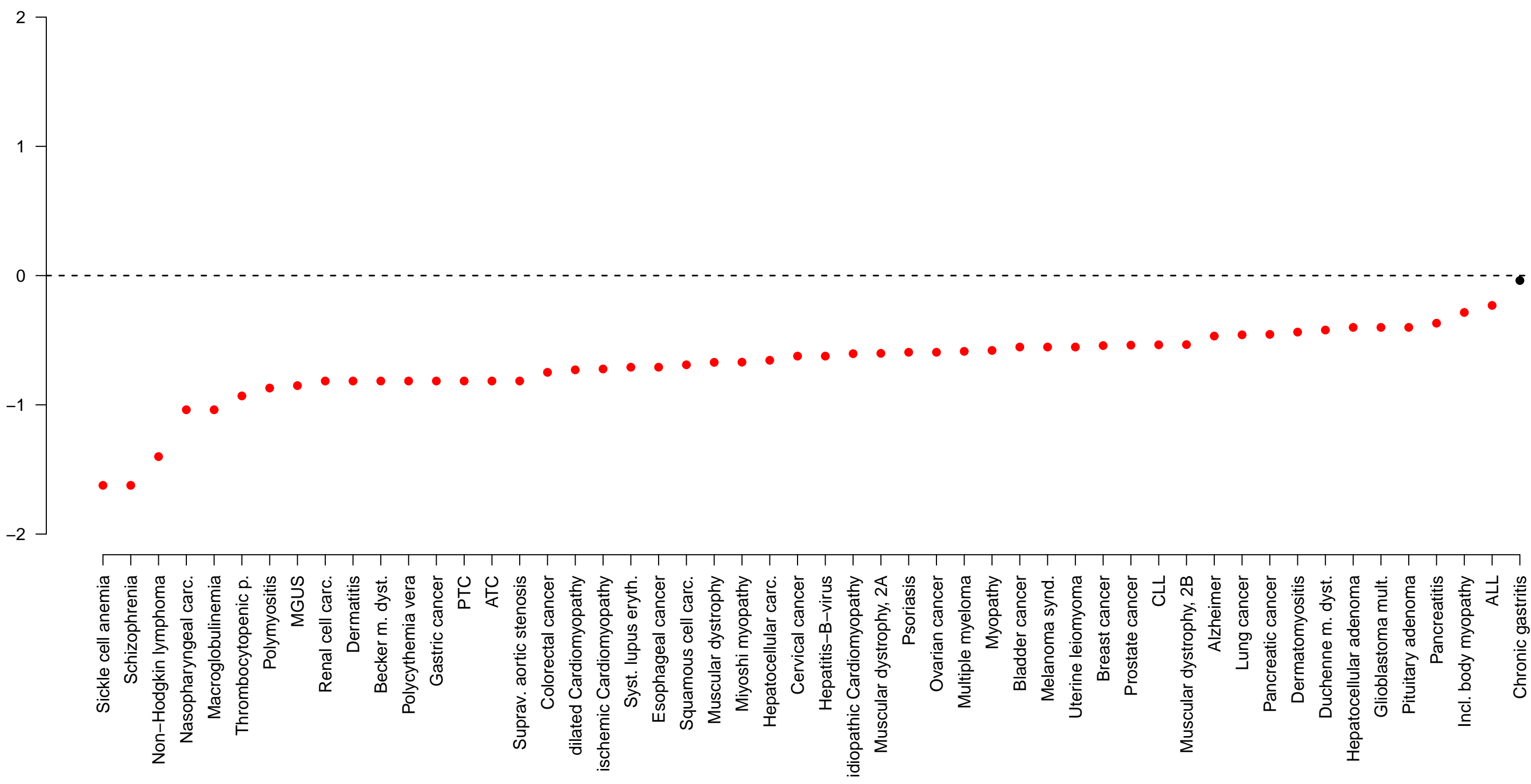

Supplement: Additional file 4 — For each disease the log-odds (LOD) score is plotted. The LOD score for disease d is given by: LODd = log2 ((yd/(xd + yd))/(yoverall/(xoverall + yoverall))) (see Materials and methods for a detailed description). We found that differentially expressed single miRNA loci are not enriched in diseases. Red points depict disorders with few deregulated single miRNA loci and black points indicate diseases without enrichment compared to random. For abbreviations of disease names see Additional file 13. [file gb-2010-11-1-r6-S4.pdf]

**A**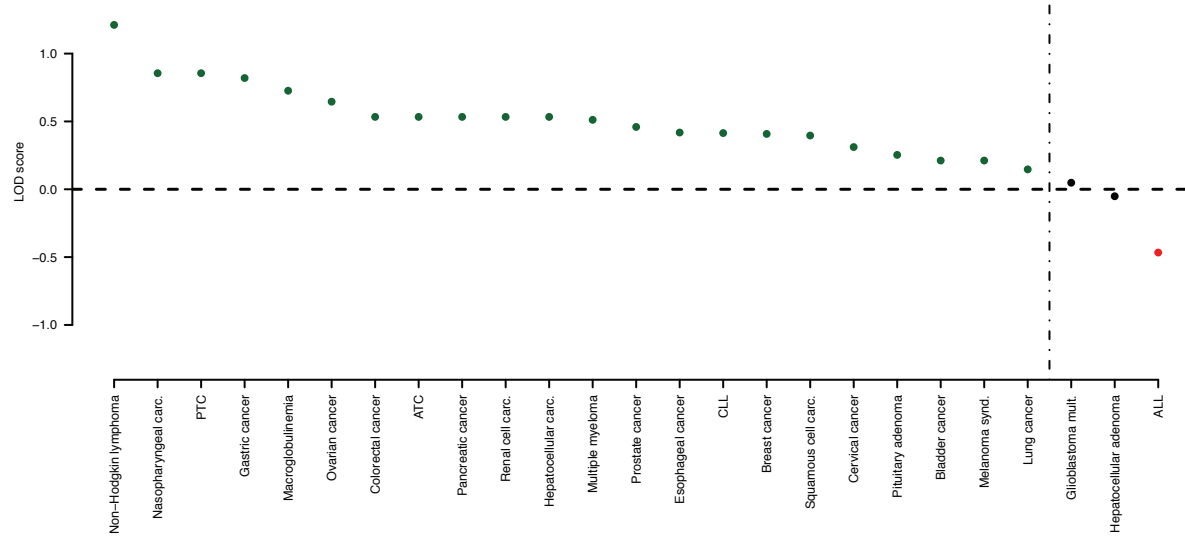**B**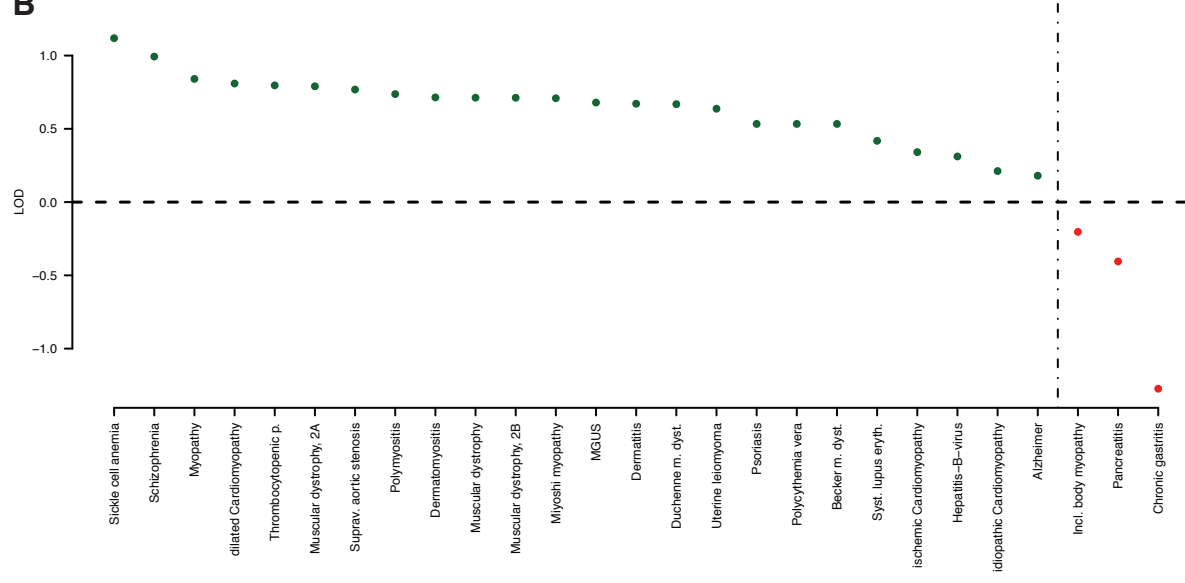

Supplement: Additional file 5 — (a) miRNA cluster enrichment for cancer diseases. (b) miRNA cluster enrichment for non-cancer diseases. For each disease the log odds (LOD) score is plotted. Green points depict enriched diseases. Black points indicate diseases without enrichment compared to random and red points depict disorders with few deregulated cluster members. [file gb-2010-11-1-r6-S5.pdf]

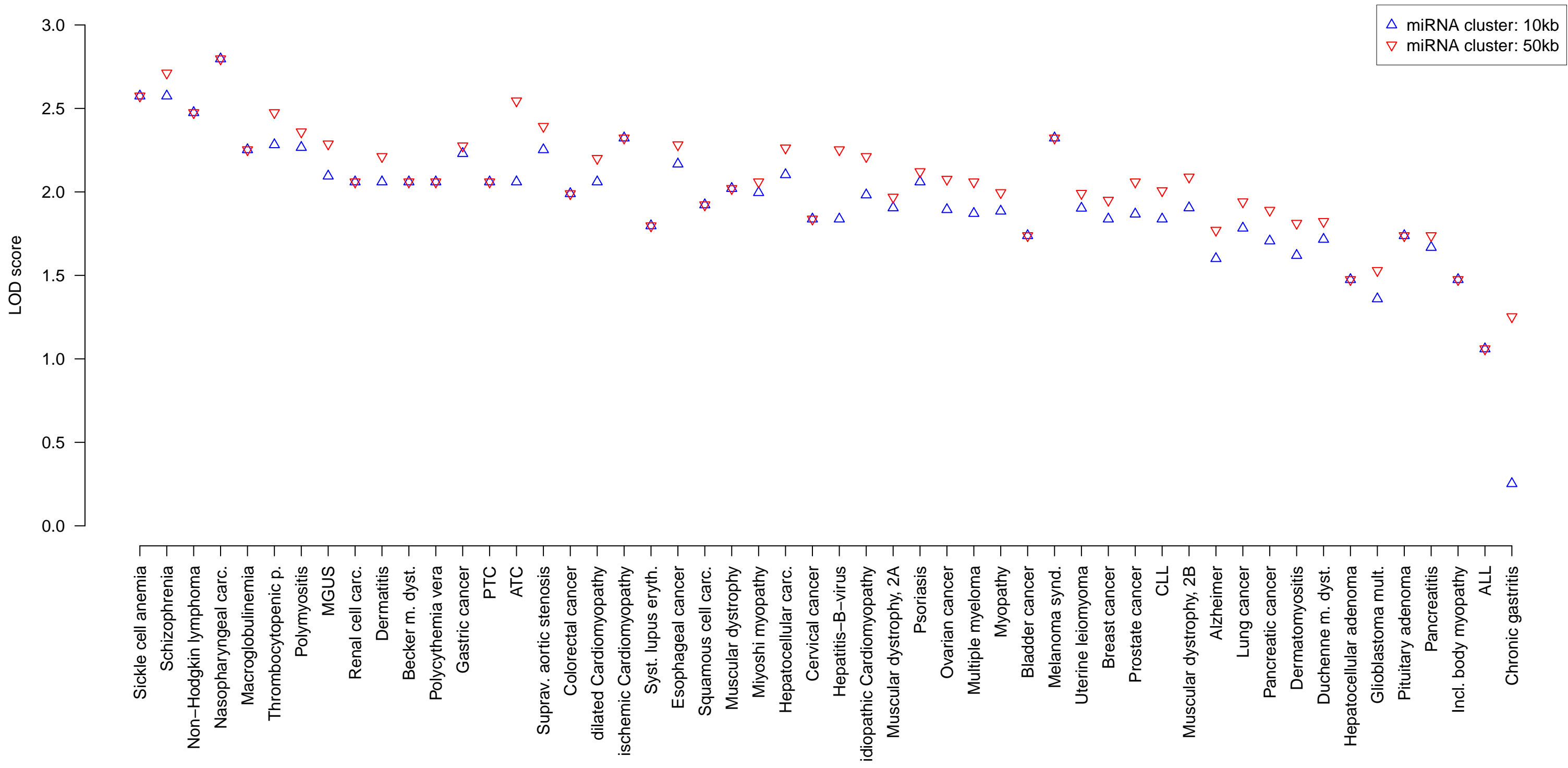

Supplement: Additional file 8 — For each disease the log odds (LOD) score is plotted. Order is based on LOD scores for polycistronic miRNA loci using a 5-kb distance threshold according to chromosomal locations. For abbreviations of disease names see Additional file 13. [file gb-2010-11-1-r6-S8.pdf]

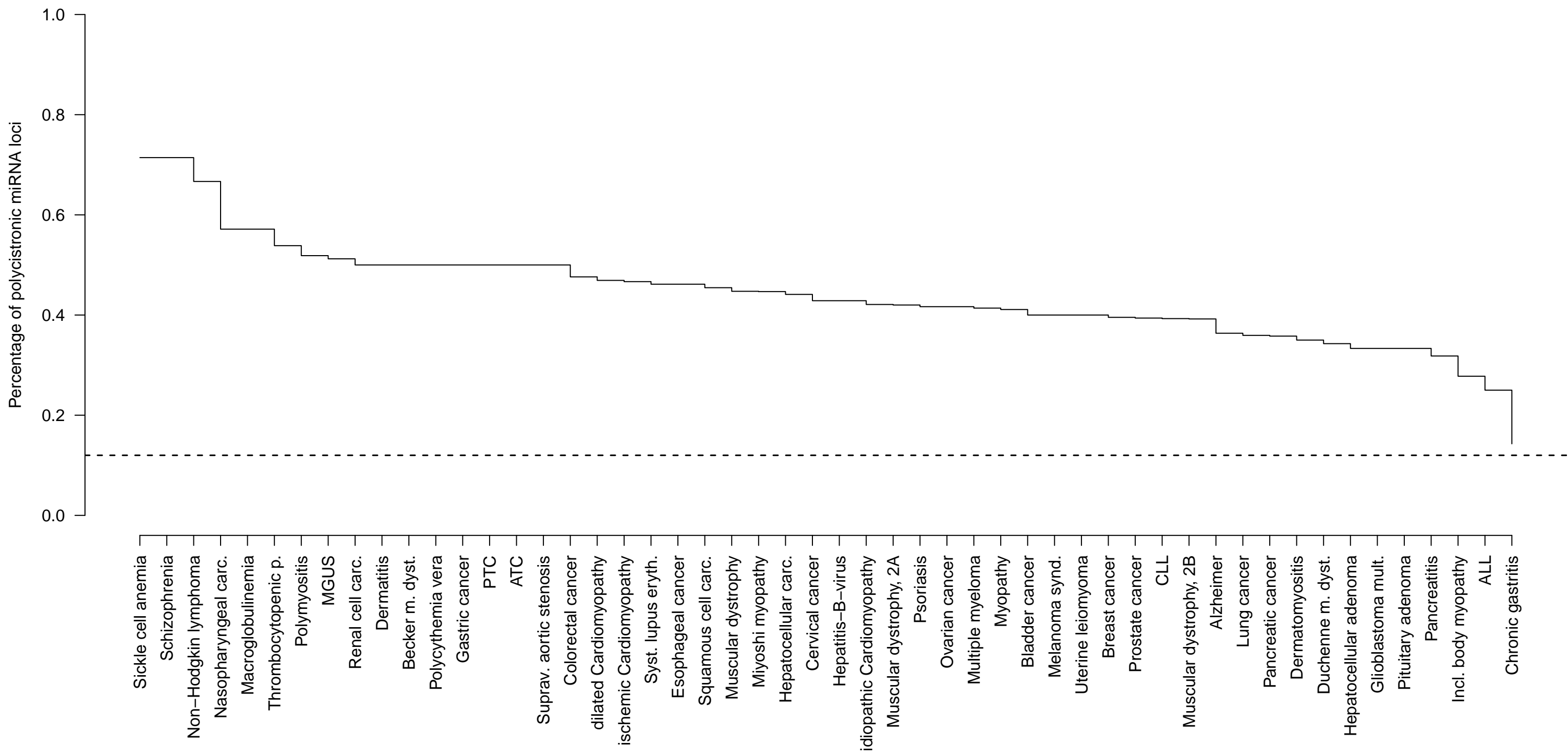

Supplement: Additional file 9 — The dashed line shows the background frequency, which is given by the number of the 62 human polycistronic miRNA loci, and the sum of polycistronic loci and the 455 human miRNAs that are not contained in any cluster as obtained from mirBase (release 12.0) using a 5-kb distance threshold according to chromosomal locations. For abbreviations of disease names see Additional file 13. [file gb-2010-11-1-r6-S9.pdf]

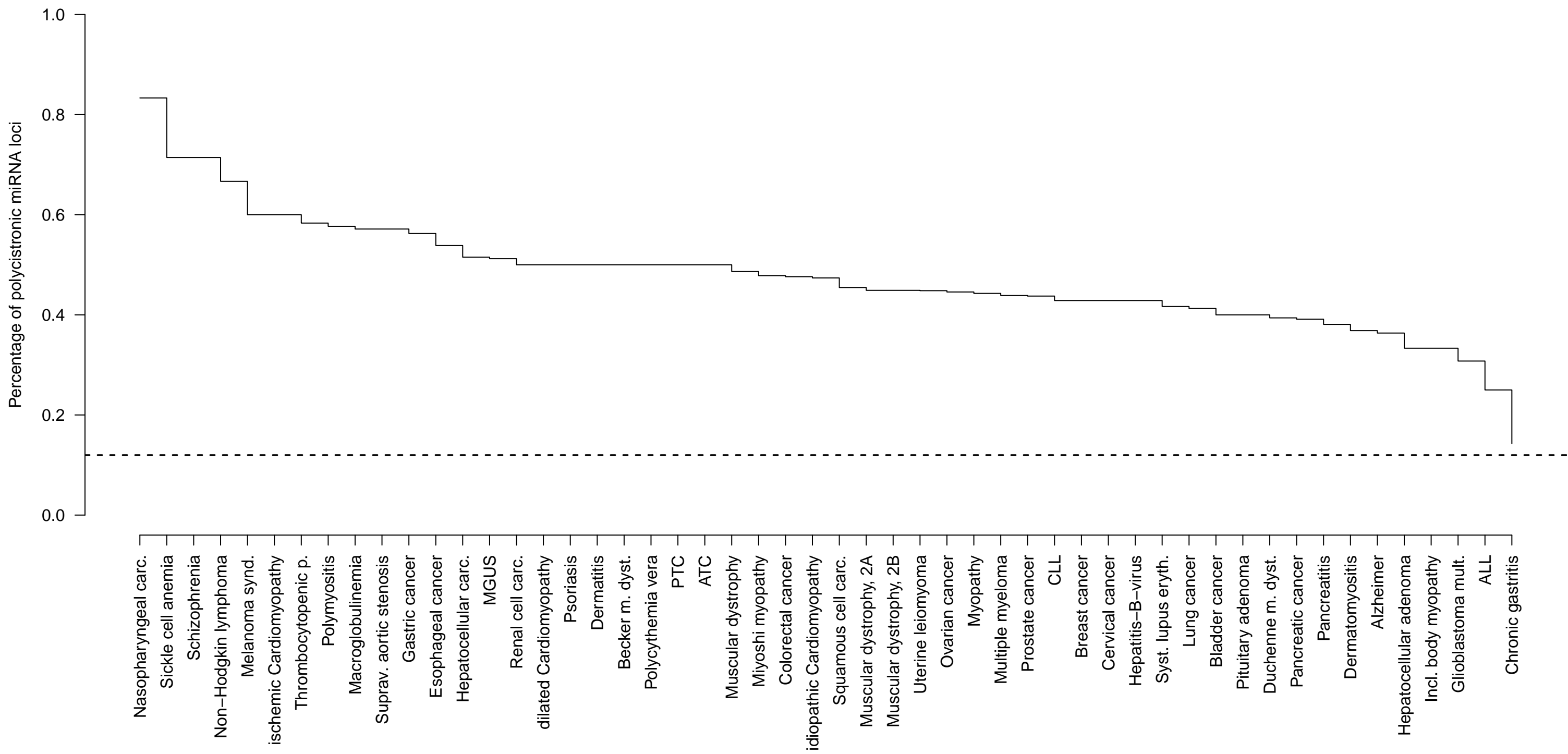

Supplement: Additional file 10 — The dashed line shows the background frequency, which is given by the number of the 65 human polycistronic miRNA loci, and the sum of polycistronic loci and the 452 human miRNAs that are not contained in any cluster as obtained from miRBase (release 12.0) using a 10-kb distance threshold according to chromosomal locations. For abbreviations of disease names see Additional file 13. [file gb-2010-11-1-r6-S10.pdf]

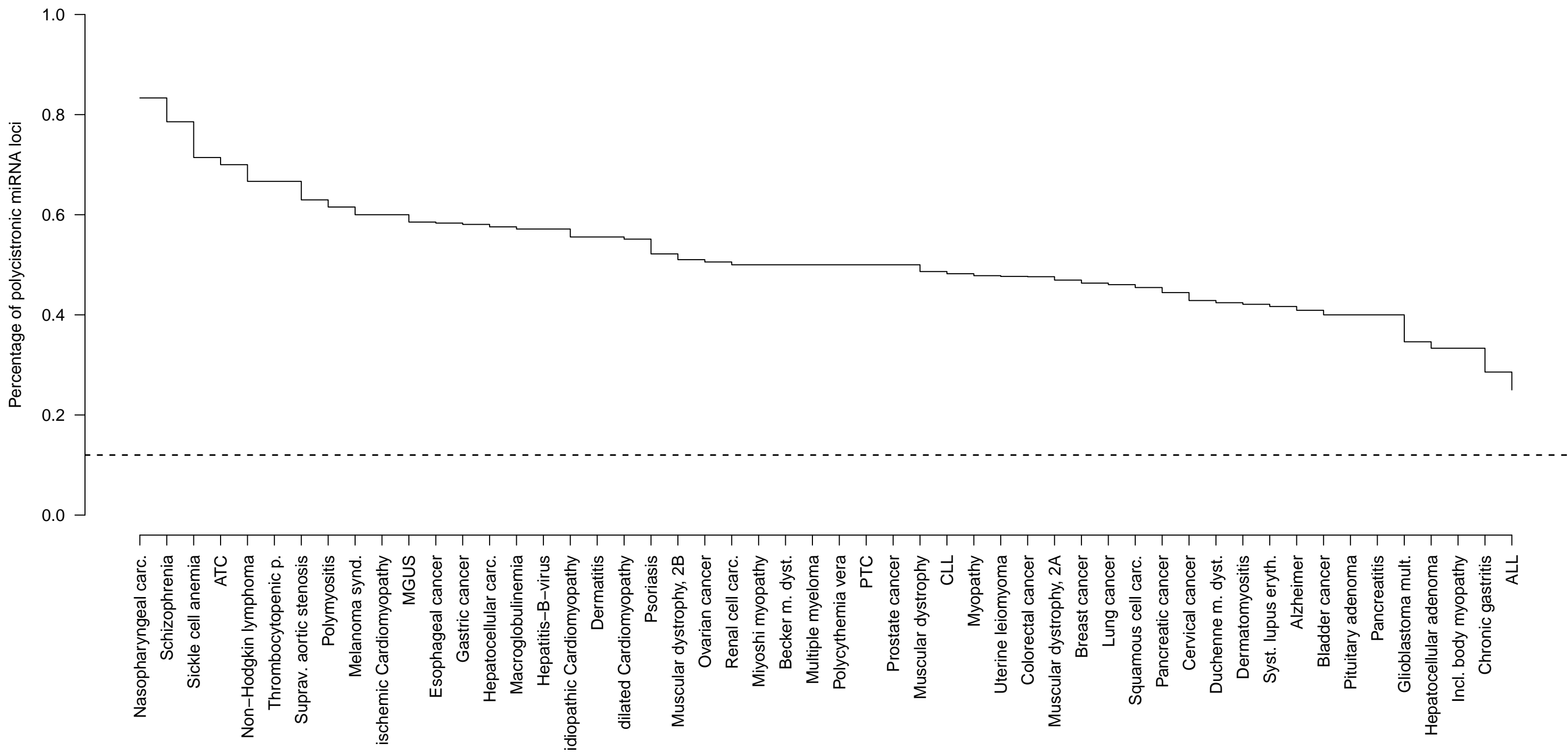

Supplement: Additional file 11 — The dashed line shows the background frequency, which is given by the number of the 72 human polycistronic miRNA loci, and the sum of polycistronic loci and the 445 human miRNAs that are not contained in any cluster as obtained from mirBase (release 12.0) using a 50-kb distance threshold according to chromosomal locations. For abbreviations of disease names see Additional file 13. [file gb-2010-11-1-r6-S11.pdf]

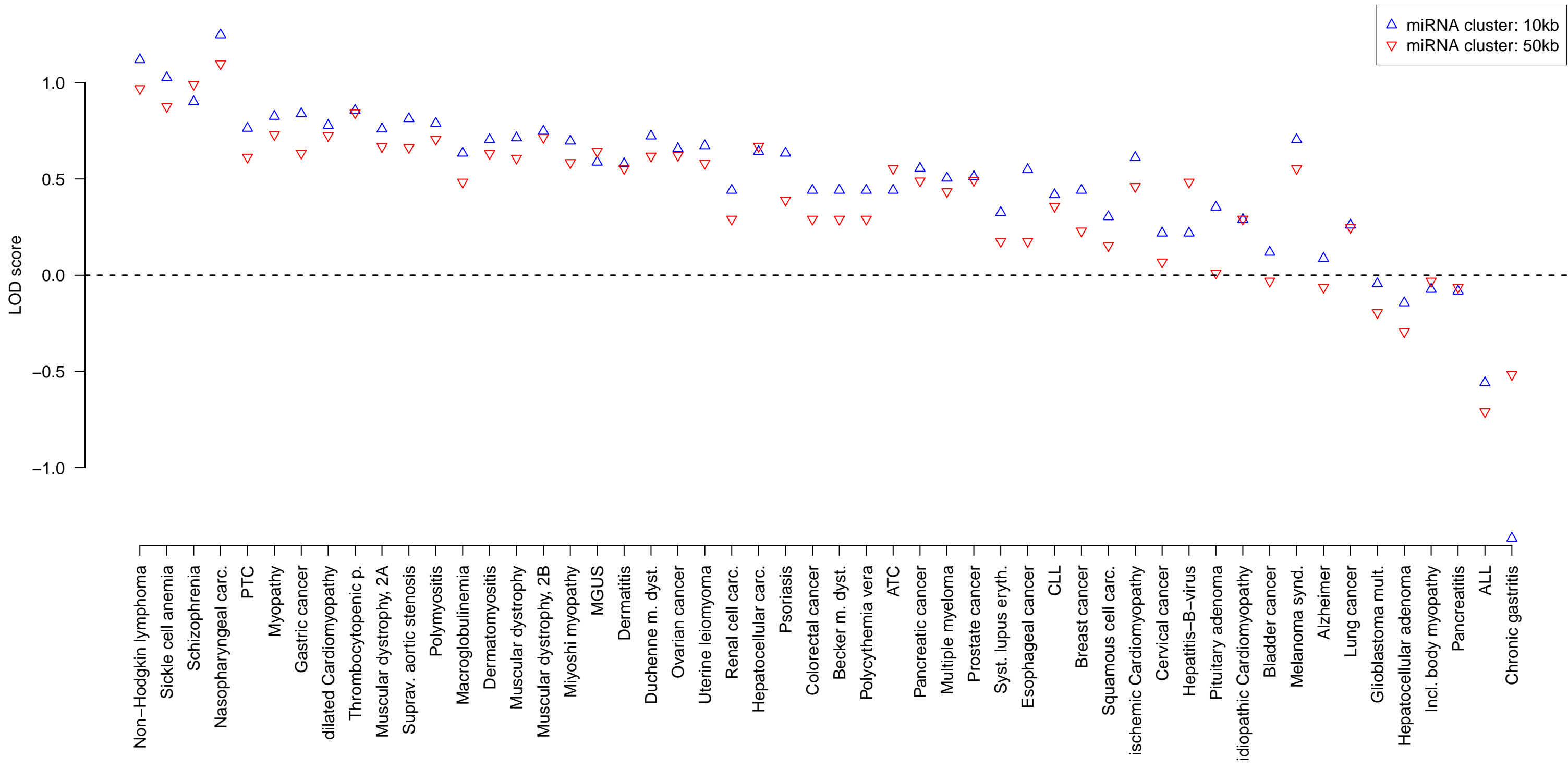

Supplement: Additional file 12 — For each disease the log odds (LOD) score is plotted. Order is based on LOD scores for miRNA clusters using a 5-kb threshold. [file gb-2010-11-1-r6-S12.pdf]
